# Epidemiology of type 1 diabetes mellitus in children and adolescents: A 50‐year, single‐center experience

**DOI:** 10.1111/1753-0407.13562

**Published:** 2024-04-25

**Authors:** Nurgun Kandemir, Dogus Vuralli, Alev Ozon, Nazlı Gonc, Didem Ardicli, Lala Jalilova, Omer Nazim Gulcek, Ayfer Alikasifoglu

**Affiliations:** ^1^ Hacettepe University Faculty of Medicine Department of Pediatric Endocrinology Ankara Turkey; ^2^ Hacettepe University Faculty of Medicine Department of Pediatrics Ankara Turkey

**Keywords:** age groups, diabetic ketoacidosis, epidemiology, Turkey, type 1 diabetes mellitus

## Abstract

**Background:**

Global variations in epidemiology of type 1 diabetes mellitus (T1DM) exist. This study is designed to examine demographic and clinical features of T1DM over the past 3 decades as well as evolving trends in epidemiology over last 50 years.

**Methods:**

Clinical characteristics of 925 patients with T1DM over last 30 years (1990–2019) were evaluated and compared to previously published data of 477 patients diagnosed between 1969 and 1990 from one of the major referral centers for diabetes in Turkey.

**Results:**

Mean age at diagnosis decreased from 9.5 ± 4.0 to 7.1 ± 3.6 years within the past 50 years (*p* < .001). Age at diagnosis peaked at 12–14 years between 1969 and 1990, then fell to 10–11.9 years between 1990 and 1999, and to 4–5.9 years between 2000–2009 and 2010–2019 (*p* = .005). Although the percentage of patients diagnosed <6 years of age is gradually increasing, the percentage between the ages of 6 and 11.9 years is decreasing, and the percentage diagnosed ≥12 years remained stable. A total of 47.5% of patients had ketoacidosis, 38.2% had ketosis, and 14.3% had only hyperglycemia. 23% of patients had severe diabetic ketoacidosis (DKA), whereas 42% had moderate. Over last 3 decades, there has been no change in frequency of ketoacidosis at presentation, but there has been significant decline in severity (*p* = .865, and *p* < .001, respectively). Although the frequency of patients with mild DKA increased over time, frequency of patients with moderate DKA decreased; however, no significant difference was observed among patients with severe ketoacidosis. DKA was more frequent and severe in patients <6 years of age (*p* = .005, and *p* < .001, respectively).

**Conclusion:**

Age at diagnosis shifted to younger ages in T1DM in the past 50 years. Half of patients had ketoacidosis at diagnosis and frequency of presentation with DKA did not decrease, but severity decreased slightly. Increase in prevalence of T1DM in the younger age group and the fact that half of patients present with DKA indicate that awareness should be increased in terms of early diagnosis and treatment.

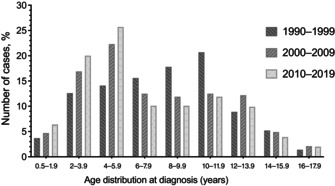

## Graphical abstract

Highlights

- This study provides important data from a single quarternary reference center where cases with type 1 diabetes mellitus (T1DM) come from a wide range of geographical distribution over 50 years and may well reflect secular trends in T1DM in Turkey.
- The mean age at diagnosis was 9.5 ± 4.0 years from 1969 to 1990, and it significantly decreased to 7.1 ± 3.6 years over the subsequent 50 years. Similarly, age at diagnosis peaked at 12–14 years between 1969 and 1990, then fell to 10–11.9 years between 1990 and 1999, and to 4–5.9 years between 2000–2009 and 2010–2019.
- Although the percentage of patients diagnosed <6 years of age is gradually increasing, the percentage diagnosed between ages of 6 and 11.9 years is decreasing, and the percentage diagnosed ≥12 years remained stable.
- Approximately half of the patients had ketoacidosis at the time of diagnosis and the frequency of presentation with DKA did not decrease, but the severity decreased slightly.
- Although the frequency of patients with mild DKA increased over time, frequency of patients with moderate DKA decreased; however, no significant difference was observed among patients with severe ketoacidosis.

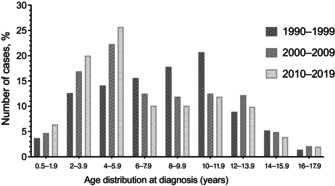

## 1 INTRODUCTION

Type 1 diabetes mellitus (T1DM), one of the most common chronic diseases of childhood, leads to autoimmune destruction of pancreatic β cells and complete insulin deficiency. T1DM is still the most frequent type of diabetes in children, despite an increase in the incidence of type 2 diabetes mellitus (T2DM) among youth in recent years.^1^, ^2^ Under the age of 19, T1DM accounts for two thirds of newly diagnosed cases of diabetes in the United States.^3^ In youth under the age of 20, T1DM accounts for 85% or more of all diabetes cases, according to global data.^2^ Epidemiological studies in recent years have revealed significant changes in the incidence and prevalence of T1DM worldwide.^4^ Age, gender, geographic region, ethnicity, and seasonal characteristics are some of the variables that affect the incidence of T1DM. It has been reported that the incidence of T1DM has increased, and diabetes occurs at earlier ages especially in the past 2 decades.^5^, ^6^ Every year, there is an increase of 3%–5% in the incidence, with younger children experiencing the greatest rise. The 0–4 age group had the largest increase in the annual incidence of T1DM, according to the EURODIAB study, which examined data from 17 European nations.^5^ In some regions, the number of newly diagnosed diabetes patients under the age of 5 is predicted to double between 2005 and 2020 if the upward trend continues.^5^ This study is designed to examine the demographic and clinical features of T1DM over the past 3 decades as well as the evolving trends in the epidemiology over the last 50 years in one of Turkey's leading diabetic centers.

## 2 PATIENTS AND METHODS

### 2.1 Study subjects

In this retrospective cross‐sectional study, demographic and clinical characteristics of children and adolescents who were followed in one of the major referral centers for diabetes in Turkey over the last 30 years (1990–2019) were evaluated. In the past, we have described the clinical and epidemiological characteristics of 477 T1DM patients diagnosed between 1969 and 1990 and 354 T1DM patients diagnosed between 1990 and 2010.^7^, ^8^ In the current study the statistical analysis of a total of 925 patients was repeated after the data from 354 patients diagnosed between 1990 and 2010 were merged with additional data from 571 patients diagnosed between 2011 and 2019. The study was approved by the Institutional Ethical Committee of Hacettepe University. Approval number: 16969557‐2201.

#### 2.1.1 The inclusion criteria

All patients followed for T1DM at a pediatric endocrinology clinic of a university hospital (single center) over the last 30 years (1990–2019) were recruited to the study. Diagnosis of T1DM was based on the classical symptoms of diabetes such as polyuria, polydipsia, and weight loss, accompanied by hyperglycemia (plasma glucose concentration ≥11.09 mmol/L or ≥200 mg/dL) and the need for long‐term insulin therapy. The diagnosis of T1DM was confirmed by the presence of antiglutamic acid decarboxylase, anti‐islet, or anti‐insulin antibodies. Patients, who tested negative for autoantibodies, but had classic signs and symptoms of diabetes, as well as a classic pattern of clinical presentation and a persistent need for exogenous insulin were also included in the study. Diabetic ketoacidosis (DKA) was defined as hyperglycemia (plasma glucose > 11.09 mmol/L or ≥200 mg/dL) with acidosis (venous blood pH <7.30 and/or serum bicarbonate (HCO3) < 15 mmol/L), ketonemia, and ketonuria. Patients with concomitant hyperglycemia, ketonemia, and ketonuria whose blood pH and HCO3 levels were >7.30 and >15 mmol/L, respectively, were considered to be in diabetic ketosis. DKA was categorized as mild (pH: 7.2–7.3 and HCO3: 10–15 mmol/L), moderate (pH: 7.1–7.2 and HCO3: 5–10 mmol/L), and severe (pH: <7.1 and HCO3: <5 mmol/L) depending on the degree of acidosis.

#### 2.1.2 Exclusion criterias

Children with T2DM, maturity‐onset diabetes in childhood, transient or stress hyperglycemia, secondary diabetes to chronic illnesses such cystic fibrosis, and children with an early onset of diabetes before 6 months of age were excluded from the analysis. Patients with missing data were also excluded from the study.

#### 2.1.3 Clinical data collection

Clinical information such as symptoms, duration of symptoms, concomitant infections, and laboratory findings at onset were collected from patient files along with demographic information about patients including age at onset, gender, season of onset, and geographic regions where they live.

#### 2.1.4 Grouping

The patients diagnosed over the last 30 years were divided into three groups based on the years of diagnosis with 10‐year intervals (1990–1999, 2000–2009, and 2010–2019). Age, gender, season, geographic distribution, prior infection history, symptoms, duration of symptoms, presence of acidosis and/or ketosis, and severity of ketoacidosis at diagnosis were compared between the 3 decades. Peak age at disease onset was determined by an analysis of frequency of patients grouped in 2‐year intervals over the age range studied. In addition, the patients were divided into three groups based on the age at diagnosis: <6 years, 6–11.9 years, and ≥12 years. The analysis was then rerun.

## 3 STATISTICAL ANALYSIS

Statistical analyses were performed using SPSS 21.0 for Windows software package (IBM Corp. Armonk, NY). Normality was tested using Shapiro–Wilks test. Categorical variables were represented as numbers and percentages, whereas continuous variables were presented as the mean and SD. To examine differences between independent groups, a one‐way analysis of variance with the post hoc Bonferroni correction was performed. A *p* value of <.05 is considered to be statistically significant. Bonferroni correction was applied for the significance level (adjusted significance value = 0.017 for three groups). The Pearson's chi square were used to examine categorical variables. Pairwise comparisons was performed by using Z‐test with the Bonferroni correction. The adjusted residual score was used to determine the cell of the contingency table made a significant contribution to the chi‐square statistics. A residual value >1.96 or <−1.96 indicated that the category made a significant contribution to the chi‐square statistic.

## 4 RESULTS

The 477 T1DM cases diagnosed between 1969 and 1990 and the 925 additional cases diagnosed between 1990 and 2019 constituted the study population. The 925 cases diagnosed between 1990 and 2019 were divided into 10‐year intervals, resulting in 135 children (70 girls), 385 children (202 girls), and 405 children (209 girls), respectively, from the years 1990–1999, 2000–2009, and 2010–2019. The mean age at diagnosis was 9.5 ± 4.0 years from 1969 to 1990, and it significantly decreased to 7.1 ± 3.6 years over the subsequent 50 years (Table 1, *p*< .001). Age at diagnosis in the study population peaked at 12–14 years between 1969 and 1990, then fell to 10–11.9 years between 1990 and 1999, and finally to 4–5.9 years between 2000 and 2009 and 2010–2019 (*p* = .005). Female to male ratio was 1 in 1969–1990, and decreased to 0.92 over the subsequent 30 years.

**TABLE 1:** Demographic characteristics of the patients with T1DM in the last 30 years.

Demographic features	Time‐frames (10‐year intervals)				
	1990–2019	1990–1999	2000–2009	2010–2019	*p* value
	All patients (n:925)	(n: 135)	(n:385)	(n:405)	
		(14.6%)	(41.6%)	(43.8%)	
Sex (Boy/girl)					.914
(n)	444/481	65/70	183/202	196/209	
(%)	48.0/52.0	48.1/51.9	47.5/52.5	48.4/51.6	
Boy/girl	0.92	0.93	0.90	0.94	
Mean age at diagnosis	7.5 ± 3.9	8.3 ± 3.9	7.7 ± 3.8	7.1 ± 3.6	<.001^a^, ^b^, ^c^
Age at diagnosis^d^					<.001
<6 years^d^	421 (45.5%)	41 (30.4%)	169 (43.9%)	211 (52.1%)	
6–11.9 years^d^	345 (37.3%)	73 (54.1%)	142 (36.9%)	130 (32.1%)	
≥12 years	159 (17.2%)	21 (15.5%)	74 (19.2%)	64 (15.8%)	
Age distribution at diagnosis (years)^e^					.010
0.5–1.9	49 (5.3%)	5 (3.7%)	18 (4.7%)	26 (6.4%)	
2–3.9^e^	163 (17.6%)	17 (12.6%)	65 (16.9%)	81 (20.0%)	
4–5.9^e^	209 (22.6%)	19 (14.1%)	86 (22.3%)	104 (25.7%)	
6–7.9^e^	110 (11.9%)	21 (15.6%)	48 (12.5%)	41 (10.1%)	
8–9.9^e^	111 (12.0%)	24 (17.8%)	46 (11.9%)	41 (10.1%)	
10–11.9^e^	124 (13.4%)	28 (20.7%)	48 (12.5%)	48 (11.9%)	
12–13.9	99 (10.7%)	12 (8.9%)	47 (12.2%)	40 (9.9%)	
14–15.9	42 (4.5%)	7 (5.2%)	19 (4.9%)	16 (3.9%)	
16–17.9	18 (2.0%)	2 (1.4%)	8 (2.1%)	8 (2.0%)	

^a^ 1990–1999 vs. 2000–2009, *p* < .001.

^b^ 1990–1999 vs. 2010–2019, *p* < .001.

^c^ 2000–2009 vs. 2010–2019, *p* < .001.

^d^ Pearson chi‐square test was used to compare three time frames (10‐year intervals) in terms of three age groups (<6 years, 6–11.9 years, ≥12 years). The age intervals marked with (d) made significant contributions to the chi‐square statistic as determined by the adjusted residual scores.

^e^ Pearson chi‐square test was used to compare three time frames (10‐year intervals) in terms of the nine age groups. The age intervals marked with (e) made significant contributions to the chi‐square statistic as determined by the adjusted residual scores.

**FIGURE 1:**
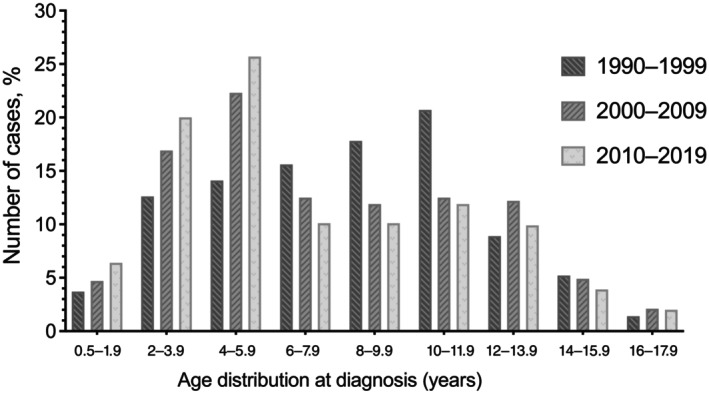
Age distribution of the patients with T1DM at diagnosis. T1DM, type 1 diabetes mellitus.

Although the percentage of patients diagnosed under the age of 6 years is gradually increasing during the past 30 years (1990–2019), the percentage diagnosed between the ages of 6 and 11.9 years is decreasing, and the percentage diagnosed at or after the age of 12 years has remained stable (Table 1). Similarly, in the last 30 years percentages of patients diagnosed between 2–3.9 and 4–5.9 years have increased, whereas the percentages of patients aged 6–7.9, 8–9.9, and 10–11.9 years have decreased (*p* = .010) (Table 1, and Figure 1).

Patients from all over the country were part of the study, with Central Anatolia accounting for the majority of them (63.8%); 15.6% of patients came from North Anatolia (Black Sea region), followed by 10.0% from Eastern and Southeast Anatolia, and the remaining 3.6%, 3.8%, and 3.2% were from Mediterranean, Aegean, and Marmara regions. Winter was the most frequent season at the time of diagnosis, followed by fall. Diagnoses were made for 33.7% of the patients in the winter, 28.1% in the autumn, 19.9% in the spring, and 18.3% in the summer. 35% of patients had infection at the time of diagnosis, upper respiratory infections was the most common (75.6%). Other infections were urinary tract infections in 15.4%, gastroenteritis in 5.6%, lower respiratory infections in 1.9%, and other types of infections in 1.5% of the patients. In the past 50 years, there had been little change in geographic distribution, seasonality, or the presence of infection during diagnosis.

The most common symptoms were polyuria (79.2%), polydipsia (79.4%), weight loss (69.5%), and polyphagia (31.8%). No matter the patient's age—<6 years, between 6 and 11.9 years, or older—the frequency of these symptoms was similar. However, frequency of enuresis nocturna and vomiting increased with younger age (39.9 and 40.1% for <6 years old, 29.9 and 29.4% for 6–11.9 years, 7.9 and 19.7% for ≥12 years, *p* < .001, and *p* < 0.001, respectively). In the last 30 years, patients diagnosed <6 years old had significantly shorter symptom duration (*p* < .001) (Table 2). Additionally, DKA was more frequent and severe in patients diagnosed before 6 years of age (*p* = .005 and *p*< .001, respectively) (Table 2).

**TABLE 2:** Comparison of the duration of symptoms and the presence of DKA by age groups between 1990 and 2019.

Clinical features	All patients	<6 years	6–11.9 years	≥12 years	*p* value
		(n:421)	(n:345)	(n:159)	
		(45.5%)	(37.3%)	(17.2%)	
Duration of symptoms^a^					<.001
≤15 days^a^	235/925 (25.4%)	135/421 (32.1%)	69/345 (20.0%)	31/159 (19.5%)	
15–30 days	362/925 (39.1%)	168/421 (39.9%)	139/345 (40.3%)	55/159 (34.6%)	
>30 days^a^	328/925 (35.5%)	118/421 (28.0%)	137/345 (39.7%)	73/159 (45.9%)	
Metabolic status at presentation^b^					.005
Presence of DKA^b^	440/925 (47.5%)	222/421 (52.7%)	155/345 (44.9%)	63/159 (39.6%)	
Presence of DK	353/925 (38.2%)	153/421 (36.4%)	132/345 (38.3%)	68/159 (42.8%)	
Hyperglycemia without ketosis or acidosis^b^	132/925 (14.3%)	46/421 (10.9%)	58/345 (16.8%)	28/159 (17.6%)	
Severity of DKA^c^					<.001
Mild DKA (%)^c^	154/440 (35.0%)	48/222 (21.6%)	80/155 (51.6%)	26/63 (41.3%)	
Moderate DKA (%)^c^	185/440 (42.0%)	107/222 (48.2%)	52/155 (33.6%)	26/63 (41.3%)	
Severe DKA (%)^c^	101/440 (23.0%)	67/222 (30.2%)	23/155 (14.8%)	11/63 (17.4%)	

^a^ Pearson chi‐square test was used to compare age groups in terms of duration of symptoms. The duration of symptoms marked with (a) made significant contributions to the chi‐square statistic as determined by the adjusted residual scores.

^b^ Pearson chi‐square test was used to compare age groups in terms of metabolic status at presentation. The metabolic status at presentation marked with (b) made significant contributions to the chi‐square statistic as determined by the adjusted residual scores.

^c^ Pearson chi‐square test was used to compare age groups in terms of severity of DKA. The severity of DKA marked with (c) made significant contributions to the chi‐square statistic as determined by the adjusted residual scores.

Abbreviation: DKA, diabetic ketoacidosis.

A total of 47.5% of the patients had ketoacidosis, 38.2% had ketosis, and 14.3% had only hyperglycemia (Table 3). Of the patients studied, 23% had severe DKA, compared to 42% who had moderate DKA and 35% who had mild DKA (Table 3). Over the past 3 decades, there has been no change in the frequency of ketoacidosis or ketosis at presentation, but there has been a significant decline in the severity of ketoacidosis (*p* = .865, and *p* < .001, respectively). Although frequency of patients with mild DKA increased over time, frequency of patients with moderate DKA decreased over the years; however, no significant difference was observed among patients with severe ketoacidosis (*p* < .001).

**TABLE 3:** DKA status of patients at admission across the decades from 1990 to 2019.

Metabolic features	1990–2019	1990–1999	2000–2009	2010–2019	*p* value
	(n:925)	(n: 135)	(n:385)	(n:405)	
Metabolic status at presentation					.865
Presence of DKA	440/925 (47.5%)	67/135 (49.6%)	192/385 (49.9%)	181/405 (44.7%)	
Presence of DK	353/925 (38.2%)	54/135 (40.0%)	135 /385 (35.0%)	164/405 (40.5%)	
Only hyperglycemia	132/925 (14.3%)	14/135 (10.4%)	58/385 (15.1%)	60/405 (14.8%)	
Severity of DKA					<.001
Mild DKA (%)^a^	154/440 (35.0%)	17/67 (25.4%)	59/192 (30.7%)	78/181 (43.1%)	
Moderate DKA (%)^a^	185/440 (42.0%)	34/67 (50.7%)	88/192 (45.9%)	63/181 (34.8%)	
Severe DKA (%)	101/440 (23.0%)	16/67 (23.9%)	45/192 (23.4%)	40/181 (22.1%)	

^a^ Pearson chi‐square test was used to compare three time frames (10‐year intervals) in terms of severity of DKA. Severity of DKA marked with (a) made significant contributions to the chi‐square statistic as determined by the adjusted residual scores.

Abbreviation: DKA, diabetic ketoacidosis; DK: Diabetic ketosis.

## 5 DISCUSSION

T1DM, which is the most frequent endocrine and metabolic disorder of childhood, tends to increase all over the world.^9^ It is reported that T1DM affects over 65 000 children worldwide each year, with the incidence rate increasing by about 3% annually.^10^, ^11^, ^12^ Epidemiological studies suggest that the incidence and prevalence of T1DM increased significantly in recent years, and it is more pronounced in children younger than 5 years old.^5^, ^6^, ^13^, ^14^, ^15^, ^16^, ^17^, ^18^

The current study involves patients from a single quarternary center located in central Anatolia, with many referrals from all over Turkey. Thus all regions may be represented, albeit more patients may be referred from underdeveloped regions. However, given that it covers a large patient population over the past 50 years, this study may well reflect secular trends in T1DM in Turkey. It shows that mean age at diagnosis decreased significantly from 9.5 ± 4.0 to 7.1 ± 3.6 years within the last 50 years. Similarly, the mean age at diagnosis of T1DM has been reported between 8.0 and 9.2 years in different countries recently.^10^, ^19^, ^20^

It is well known that T1DM has a bimodal age distribution. One peak between 4 and 6 years attributed to frequent exposure to infections during preschool years, and the second peak between 10 and 14 years related to the impact of anti‐insulin hormones like sex steroids, growth hormone, and psychological stress at puberty.^1^, ^21^, ^22^ In our epidemiological study including data of 1969–1990, the peak age for the diagnosis of T1DM was 12–14 years of age. It decreased to 10–12 years in the period of 1990–1999 and to 4–6 years of age in the last 2 decades (in the period of 2000–2009 and 2010–2019). A second peak appeared at 2–4 years of age. Current study from Turkey reveals a secular trend for the peak age of diagnosis toward younger ages especially in the past 30 years. International studies such as EURODIAB and DIAMOND (Diabetes Mondiale) also showed similar epidemiological change in age at onset of T1DM.^5^, ^10^, ^11^ Data from 17 European countries were evaluated in the EURODIAB study, and the incidence of T1DM was reported to be 5.4% in the 0–4 age group, 4.3% in the 5–9 age group, and 2.9% in the 10–14 age group.^5^ It is predicted that the number of newly diagnosed T1DM cases under the age of 5 will double between 2005 and 2020 in some regions of Europe and the prevalence of T1DM under the age of 15 will increase by 70%.^5^ In a study performed in Philadelphia, it was shown that the incidence of T1DM in children under the age of 5 increased by 70% between 1985 and 2004.^23^ In a study from Colorado, the highest increase in incidence was observed in the 0–4 age group.^16^ A similar sharp increase in the incidence of T1DM in children aged 0–4 years was also found in data from Israel and Finland.^24^, ^25^

It is noteworthy that this increase in the incidence of T1DM, especially in the younger age group, has become more pronounced since the mid‐1990s.^26^ In a study from Austria, although there was no change in the incidence of T1DM under the age of 5 between 1979 and 1994, it was reported that there was an increase of 9.2% per year between 1995 and 2005.^27^ Similarly, although the incidence rates were stable between 1988 and 1993 in France, a rapid increase was reported between 1994 and 1997, especially in the younger age group.^28^ However, this trend in the earlier shift of the age of onset was not demonstrated in all populations. Studies from China showed that although there is an increase in the incidence of T1DM generally, this increase is mostly due to the increased incidence in older children, and a similar incidence increase in early childhood period has not been demonstrated.^29^ A Swedish study reported an increase in age at diagnosis in children born after 2000.^30^ Similarly, an increase in age at diagnosis was reported in a study from New Zealand.^31^ In some populations, the incidence of T1DM and the age of diagnosis appear to plateau. For instance, according to the data of the US Military Health System, which includes the data of 5616 cases with T1DM under the age of 18, the prevalence (1.5/1000) and incidence (20.7–21.3/100000) of T1DM plateaued between 2002 and 2007.^32^ The incidence was higher in the 10–14 age group (from 30.9/100000 in 2008 to 35.2/100000 in 2011). A study from Australia showed a similar plateau plot in the incidence of T1DM after the peak in 2003.^33^

The increase in incidence of T1DM in younger population is an alarming situation because diabetes management is more challenging in this younger age group. Physiological characteristics, such as increased insulin sensitivity and potentially shortened honeymoon period, as well as the cognitive, behavioral, social, and emotional development in this age group may complicate daily management of T1DM. Data from the T1DM Exchange study show that 36% of children under the age of 6 fail to meet the glycemic control targets (HbA1c <8.5%) set by the American Diabetes Association and 73% fail to meet International Society for Pediatric and Adolescent Diabetes targets (HbA1c <7.5%).^34^

The exact cause of this increase is not completely understood yet. Various factors may play a role in this increase including environmental triggers, ethnic differences, genetic susceptibility, and the ability of health systems to diagnose new cases.^35^ Because genetic alterations do not take place in such a short time, environmental factors and variations caused by gene–environment interactions are thought to be the reason.

The reason for the rapid increase in the young age group may be due to the fact that this age group carries more diabetes‐prone human leukocyte antigen genes, as well as the change in environmental factors over time and the increase in disease penetration.^36^ Various gene–environment interactions are thought to initiate or accelerate autoimmune β cell destruction.^37^ The rapid increase in the incidence of T1DM in some populations supports the role of environmental factors rather than genetic predisposition. For example, the incidence of T1DM in Kuwait has increased from <4 per 100 000 to 20 per 100 000 since the end of the first gulf war in 1991.^38^ The difference in the incidence of T1DM in genetically similar but socioeconomically different populations such as Finland and Russia indicates the importance of environmental factors.^39^

Several hypotheses have been proposed regarding an earlier shift in the age of onset of diabetes. The first of these is that in children <4 years of age, the rate of progression from islet autoimmunity to clinical diabetes has been thought to be faster in recent cohorts compared to previous cohorts, although both cohorts had similar prevalence of islet autoantibodies.^40^ However, the reason for this rate of progression to clinical diabetes in children under the age of 4 is not clear. Recently, it has been questioned whether overweight and obesity, which are increasingly seen in childhood period, contribute to the increase in the incidence of T1DM and the development of T1DM at a younger age. Some studies have suggested a relationship between increased body weight and an increase in the incidence of T1DM in children and adolescents.^41^ This hypothesized relationship is called the accelerator hypothesis. The argument of those who say that there is such a relationship is that in countries where the incidence of T1DM increases, especially in young age groups, the frequency of obesity increases simultaneously in the general child population. According to the accelerator hypothesis, excessive body weight in a child who is genetically predisposed to the development of T1DM accelerates the β‐cell destruction process, causing a significant insulin deficiency to occur earlier.^41^, ^42^, ^43^ Therefore, environmental pressure from changing lifestyle habits suggests that a child with a higher body mass index (BMI) will have an accelerated β‐cell decline and the onset of diabetes will occur at a younger age.^41^

There might be two approaches to the question whether obesity is the reason or not for the increased incidence of T1DM in younger age groups. The indirect approach is to evaluate whether there is an increasing trend in the prevalence of obesity in preschooler populations in areas where incidence of T1DM is increasing. In different studies, the prevalence of overweight in children has been reported to vary between 30% and 37.3% and the prevalence of obesity between 10.2% and 13.8%.^44^ According to the results of the US National Nutrition and Health Survey in 2017–2018, the prevalence of obesity in children aged 2–5 years was 13.4%, and it was 20.3% and 21.2% in children aged 6–11 years and adolescents aged 12–19, respectively.^45^ The prevalence of obesity in children and adolescents with T1DM is also increasing parallel to the general population.^46^, ^47^, ^48^ Previous research from various regions of the world revealed that 25%–38.5% of children with T1DM had overweight and obesity.^47^, ^49^, ^50^ However there are no data suggesting that obesity increases especially in the younger age groups. In fact it is well known that T2DM that is more closely related to obesity is seen after 10 years of age.^51^ In Turkey, data on the prevalence of overweight and obesity both in diabetic and nondiabetic children are regional rather than nationwide. These regional studies mainly involve school‐aged children, and overweight/obesity prevalence was reported to be between 16.8% and 25.1%.^52^, ^53^, ^54^ Unfortunately data on younger age groups are quite scarce. The single most important study evaluating the prevalence of overweight/obesity in preschoolers in Turkey derives its data from the Demographic and Health Surveys Program (a regular survey carried out in more than 90 countries worldwide every 5 years). In that study, the prevalence of overweight increased from 5.3% to 11.6% and that of obesity increased from 1.3% to 2.8% in children <5 years of age during a period of 25 years.^55^

The second approach might be more direct, and it involves analyzing the correlation between BMI of those who develop T1DM and age at diagnosis. Some studies supported that obesity may play part, showing a significant inverse correlation between BMI and age at diagnosis.^56^, ^57^, ^58^ Although Islam et al similarly showed that higher BMI SD score was associated with younger age at diagnosis, they also showed the prevalence of overweight and obesity at diagnosis has not been changed in children with T1DM over 15 years.^59^ These findings suggest that obesity alone cannot explain the increasing incidence of T1DM in younger age group observed recently. In addition, there are many other studies that found no association between BMI SDS, age at diagnosis and incidence of T1DM, thus, the accelerator hypothesis is not universally accepted.^31^, ^60^ Although the BMI of the patients at the time of diagnosis is not examined in the current study, we cannot evaluate the role of obesity in this shift, in the age of onset of T1DM to younger ages. This was an important limitation of the current study.

Other hypotheses that are put forward as a possible explanation for the increased incidence of T1DM in young children include the hygiene hypothesis and early exposure to various environmental factors. The hygiene hypothesis proposes that reduced exposure to infectious agents at a young age results in immaturity of the immune system and thus an increase in immune‐modulated diseases.^61^ The increase in vitamin D deficiency and the increase in early exposure to cow's milk are other environmental factors that have been put forward to explain the increase in T1DM in young children.^62^, ^63^

The current study involves data from a single center and does not represent the whole country. However, our clinic is in the middle of Anatolia, working as a major referral center accepting patients from all regions. Thus, the current data may reflect general trends over time with limitations, until nationwide studies can be conducted. Other studies from Turkey reflect cross‐sectional incidence or prevalence data. There is only one nationwide study in Turkey performed until now that reports incidence and prevalence of T1DM.^64^ It reported a prevalence of 0.75/1000 over a period of 3 years (2011–2013) and an incidence of 10.8/100000 for the year 2013. However, it achieved records from the National Social Security Registry, and because the authors have stated that these records were not confirmed, the numbers may be overestimations. Other studies provide regional data about incidence and prevalence of T1D, and the incidence was 8.99/100000 in Northwest region, 9.14/100000 in Southeastern region, 13.1/100000 in Eastern Anatolian region, and the prevalence was 0.27/1000 in Ankara, 0.67/1000 in Istanbul, and 0.42/1000 in Southeastern Anatolian region.^65^, ^66^, ^67^, ^68^, ^69^, ^70^ However, as stated previously, these studies do not involve longitudinal data.

Although most autoimmune diseases are more common in girls, T1DM in children and adolescents affects boys and girls equally.^71^ Although the boy/girl ratio was 1 in the period of 1969–1990, this ratio decreased to 0.92 in the last 30 years in our study. Although T1DM is more common in boys in some European countries with a high incidence of T1DM (eg, Sardinia in Italy, the United Kingdom, Norway, Sweden, and Finland), T1DM is more prevalent in girls in non‐European populations with a low incidence of T1DM.^72^, ^73^ In some studies performed in boys aged 13 and over of European origin, it was reported that they developed T1DM more frequently than girls in the same age group and same geographical region, and the boy/girl ratio was 3:2 for this age group.^25^, ^74^ The same boy/girl ratio was reported for children under 6 years of age in an observational study from Massachusetts.^75^ In Denmark, France, and the Netherlands, the boy/girl ratio was reported to be equal to one.^73^, ^74^

There are many studies showing that T1DM is diagnosed more in the autumn and winter.^76^, ^77^, ^78^, ^79^ Moltchanova et al, using the data of the international DIAMOND study carried out by the World Health Organization, reported that a significant difference was found in children with T1DM under the age of 14 diagnosed between 1990 and 1999 in terms of seasons at diagnosis, with the highest diagnosis in October–January, and the lowest in June–August.^76^ Similar to other studies, the frequency of T1DM in the current study was higher in cold months such as winter and autumn. Similarly, in the previous epidemiological study performed in our center, it was shown that patients were diagnosed with T1DM most commonly in the winter season. One opinion that explains the more frequent occurrence of T1DM in the cold months is that viral infections are more common in winter and autumn, and infections are thought to trigger autoimmune β‐cell destruction. One third of the patients in our study had infection at the time of diagnosis, and when the seasons at diagnosis of these patients were considered, they were mostly diagnosed in winter and autumn. This supports the argument that viral infections have an effect at the season of diagnosis. In the previous study performed in our center, it was reported that approximately half of the patients had an infection at the time of diagnosis, and these patients were especially in the 0–6 age group.

Another thought put forward to explain the relationship between T1DM and the seasons is the protective effect of sunlight and vitamin D. In an animal study by Sloka et al, it was shown that there is a negative correlation between daily ultraviolet‐B (UVB) radiation and the incidence of T1DM.^80^ It has been suggested that the lower incidence of T1DM in summer months compared to winter months may be due to higher UVB radiation exposure. In the study of Douglas et al, in which they examined the effect of diet and exercise on the development of T1DM, it is suggested that spending more time at home in front of television during the winter months may be effective in the onset of diabetes and that increased daily activity in the summer may also be protective.^81^

The symptoms of diabetes did not vary over the years.^82^, ^83^ The most common complaints of the patients were polyuria and polydipsia, followed by weight loss. Enuresis nocturna and vomiting were the first complaints in a substantial portion of the patients, such as one third, and the frequency of these symptoms increased as the age at diagnosis decreased. Duration of symptoms was shorter than 1 month in two thirds of the patients, and this duration was shorter than 15 days in one fourth of them. The duration of symptoms in the cases diagnosed at a younger age was significantly shorter, and the prevalence and severity of DKA were higher.

DKA is one of the most serious complications of T1DM causing morbidity and mortality. In the current study about half of the cases still present with DKA, this ratio did not change over the 50 years. The frequency of DKA in the current study was found to be quite high compared to countries in the world where T1DM is common. The frequency of DKA at the time of diagnosis in children with T1DM varies from 15% to 67% in literature.^84^ According to the EURODIAB study, there is an inverse relationship between incidence rates of T1DM and the frequency of admission with DKA. It has been reported that in countries with low incidence, 60% of patients present with DKA at the time of diagnosis, whereas this rate is around 15%–20% in populations where T1DM is common, patients presenting earlier due to increased social awareness.^85^, ^86^ Although the frequency of admission with DKA is around 15%–20% in countries such as Sweden and Canada, it reaches up to 70% in African countries. The frequency of DKA was found to be 55% in Saudi Arabia, 37% in Austria, and 19.4% in Finland.^87^, ^88^, ^89^ Considering the frequencies of DKA in other studies performed in our country, the frequency of DKA was found as 33% in Bursa, 41% in the Western Black Sea region, 45% in Elazig, and 65% in the Eastern Anatolia region.^90^, ^91^, ^92^, ^93^

In the current study, more than half of the cases diagnosed under the age of 6 were admitted with DKA and one third with ketosis, whereas only 10% presented with hyperglycemia without ketosis and acidosis. The frequency of DKA and ketosis was also high in the 6–12 age group, frequency of DKA being 45% and that of ketosis being 38% in this age group. In children older than 12 years of age, the frequency of DKA and ketosis were similar to each other, and both were around 40%. In this age group, admission with only hyperglycemia was significantly higher than the other two age groups. In addition, apart from the frequency of DKA, the severity of DKA was more severe in cases diagnosed under the age of 6 years. Similarly, DKA was found to be more common in children under the age of 6 or in societies with low socioeconomic status in literature. Complaints in young children cannot be noticed by families and health personnel, and the cases present with more severe ketoacidosis due to metabolic decompensation.^94^ In a study from England, it was reported that diabetic children of Asian origin under the age of 5 were more likely to present with DKA than non‐Asian children (68% vs 32%).^95^ In the study performed by Schober et al. in Austria, the prevalence of DKA under the age of 15 was 37.2%, whereas it was reported that 60% of children under the age of 2 presented with DKA.^88^

Several studies showed the incidence of DKA at diagnosis decreased over time due to the increased awareness of families and clinicians.^96^ However, in some populations, similar to the current study, it has been shown that the frequency of DKA does not decrease over time. In a study from Austria, there was no change in the frequency of admission with DKA in the last 20 years.^88^ In a study from Finland, it was found that although the rate of DKA at diagnosis decreased in the last 10 years, whereas an increase was observed in children under 5 years old (especially under 2 years old).^89^ Although the frequency of DKA remained constant over time in our study, the severity of ketoacidosis decreased significantly. Although the frequency of patients with mild DKA increased over time, frequency of patients with moderate DKA decreased over the years, however, no significant difference was observed among patients with severe ketoacidosis. The fact that frequency of ketoacidosis did not decrease even though awareness of diabetes increased may be related to the shift of onset of diabetes toward younger ages.

In conclusion, this study provides important data from a single reference center where cases with T1DM coming from a wide range of geographical distribution over 50 years. We have showed that age of diagnosis shifted to younger ages in the past 50 years in our country similar to the other European countries where the incidence and prevalence of T1DM is high. The reason for this increased incidence in children <5 years of age, which is observed in most of the countries that have high prevalence of T1DM but not in countries with a lower prevalence such as China, is unclear. In addition, approximately half of the patients who applied to our center had ketoacidosis at the time of diagnosis and frequency of presentation with DKA did not decrease in the last 30 years, but the severity decreased slightly. Increase in the prevalence of T1DM in the younger age group and the fact that half of the patients still present with the DKA indicate that awareness of diabetes should be increased in our community in terms of early diagnosis and treatment. Search for risk factors that may be associated with the overall increased incidence of T1DM and the marked increase in its incidence in young children should be continued. Maintaining and improving surveillance systems that will help to elucidate the etiology of this worrying worldwide trend in childhood T1DM is necessary.

## AUTHOR CONTRIBUTIONS

Nurgun Kandemir and Dogus Vuralli conceptualized and designed the study; contributed to acquisition, analysis, and interpretation of data; and wrote the manuscript. Alev Ozon, Nazlı Gonc, and Ayfer Alikasifoglu contributed to analysis and interpretation of data and contributed to manuscript writing and checking. Didem Ardicli, Lala Jalilova, and Omer Nazim Gulcek contributed to acquisition and analysis of the data. All authors have accepted responsibility for the entire content of this submitted manuscript and approved the final version of the manuscript.

## FUNDING INFORMATION

No financial assistance was obtained for this study.

## DISCLOSURE

The authors have no conflicts of interest to disclose.
